# Possible expansion of *Ixodes ricinus* in the United Kingdom identified through the Tick Surveillance Scheme between 2013 and 2020

**DOI:** 10.1111/mve.12612

**Published:** 2022-10-14

**Authors:** Sara L. Gandy, Kayleigh M. Hansford, Jolyon M. Medlock

**Affiliations:** ^1^ Medical Entomology & Zoonoses Ecology UK Health Security Agency Salisbury UK; ^2^ NIHR Health Protection Research Unit in Environmental Change and Health London UK

**Keywords:** *Borrelia burgdorferi* s.l, distribution, surveillance, vector, vector‐borne disease

## Abstract

The tick *Ixodes ricinus* (Ixodida: Ixodidae, Linnaeus) is the main vector of several pathogens including *Borrelia burgdorferi* s.l. (agent of Lyme borreliosis) and tick‐borne encephalitis virus. Its distribution depends on many factors including suitable habitat, climate and presence of hosts. In this study, we present records of *I. ricinus* bites on humans, dogs (*Canis lupus familiaris;* Carnivora: Canidae, L.) and cats (*Felis catus*; Carnivora: Felidiae, L.) in the United Kingdom (UK) obtained through the Tick Surveillance Scheme between 2013 and 2020. We divided the UK into 20 km x 20 km grids and 9.2% (range 1.2%–30%) of grids had at least one record every year since 2013. Most regions reported a yearly increase in the percentage of grids reporting *I. ricinus* since 2013 and the highest changes occurred in the South and East England with 5%–6.7% of new grids reporting *I. ricinus* bites each year in areas that never reported ticks before. Spatiotemporal analyses suggested that, while all regions recorded *I. ricinus* in new areas every year, there was a yearly decline in the percentage of new areas covered, except for Scotland. We discuss potential drivers of tick expansion, including reforestation and increase in deer populations.

## Graphical abstract

- When dividing the United Kingdom (UK) in 20 × 20 km grids, 9.2% of grids reported at least one *Ixodes ricinus* bite every year since 2013. Most regions in the UK reported a yearly increase in the percentage of grids reporting tick bites since 2013. South and East England reported the highest increase with 5%–6.7% of new grids reporting tick bites each year in areas that never recorded *I. ricinus* before.

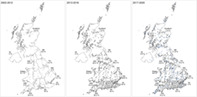

## INTRODUCTION

Vector‐borne diseases, which rely on living organisms to transmit pathogens from one host to another, represent 17% of all infectious diseases in humans, causing more than 700,000 deaths per year worldwide (WHO, 2020). The emergence of vector‐borne diseases depends on a multitude of factors, which include expansion of arthropod vectors and vector host distributions and can be due to environmental changes such as habitat fragmentation and urbanization (Gibbs et al., 2006; Parrish et al., 2008). In the Northern hemisphere, tick‐borne disease incidence has increased over recent decades and represents an ongoing burden for public health with diseases such as tick‐borne encephalitis (TBE), Lyme borreliosis, human granulocytic anaplasmosis (HGA) or Crimean‐Congo haemorrhagic fever (CCHF). Domestic animals also suffer from tick‐borne diseases, which include babesiosis and tick‐borne fever. In Europe, several tick species transmit pathogens; for instance, *Hyalomma marginatum* (Ixodida: Ixodidae, Koch) can transmit CCHF virus, *Dermacentor reticulatus* (Ixodida: Ixodidae, Fabricius) is the main vector for canine babesiosis and *Haemaphysalis punctata* (Ixodida: Ixodidae, Canestrini & Fanzago) can transmit causative agents of babesiosis and theileriosis (Földvári et al., 2016; Garcia‐Sanmartin et al., 2008; Hoogstraal, 1979; Medlock et al., 2018). However, the main vector of tick‐borne diseases in Europe is *Ixodes ricinus*, which can transmit the causative agents of Lyme borreliosis, TBE, babesiosis and HGA (Benda, 1958; Burgdorfer et al., 1985; Movila et al., 1980). *I. ricinus* is also the most widespread tick in Europe and thus, it is important to monitor potential distribution changes and understand drivers of geographical expansion in order to raise awareness of tick‐borne disease risk among the public and clinicians.

In Europe, *I. ricinus* larvae and nymphs tend to feed on small sized vertebrates (small mammals, birds) while adult females generally feed on large herbivores. Deer are important hosts for all tick life stages, are known to drive tick abundance and can facilitate tick expansion through the active dispersal of ticks (Gandy et al., 2021; Gilbert et al., 2012). *I. ricinus* are adapted to certain environmental conditions and their range is constrained by both host presence and microclimate, so geographical expansion is likely to be due to anything that can impact these components. For instance, environmental changes such as milder winters and increases in temperatures are thought to have contributed to the expansion of *I. ricinus* in colder climates and at higher altitudes (Danielová et al., 2010; Jaenson et al., 2012; Jensen et al., 2000; Lindgren et al., 2000; Lindgren et al., 2006; Medlock et al., 2013). Land use changes and reforestation might have led to an increase in deer densities, providing suitable tick hosts (Medlock et al., 2013) and the reduction in predators across Europe might also have contributed to a change in host community composition (Levi et al., 2012). Thus, expansion in the range of *I. ricinus* is likely to be a combination between changes in temperature, rainfall, forest and land use management and wildlife densities and management (Medlock et al., 2013). While increases in temperatures might lead to an expansion in colder climate, it could also lead to a decrease in *I. ricinus* presence or density in warmer countries at the limit of their geographical range (Porretta et al., 2013). Changes in human behaviour, such as a rise of outdoor activities, might also increase contact rates between ticks and humans (Hall et al., 2017).

As the incidence of tick‐borne disease is directly linked to the geographical distribution of key vector species, it is important to conduct surveillance to detect potential changes over time. In this study, we aimed to use data collected through the UK Tick Surveillance Scheme (TSS), which encourages members of the public, veterinary practices, wildlife charities and health professionals to send ticks found in the UK, to identify potential changes in *I. ricinus* distribution. Previous studies have already shown how the distribution of two other vectors (*D. reticulatus* and *H. punctata*) is changing in the UK (Medlock et al., 2017; Medlock et al., 2018; Phipps et al., 2020). This study focuses on *I. ricinus* and, whilst a previous study assessed the potential change in *I. ricinus* in the UK using TSS data alongside historical Biological Records Centre data (Cull et al., 2018), our current study uses TSS data only from humans, cats and dogs and includes new data from 2016 to 2020. The aim of this study is to provide some quantitative and spatial assessment of possible changes in *I. ricinus* distribution in the UK using a long‐term dataset that could be used to increase the prioritization of interventions.

## MATERIALS AND METHODS

### Ixodes ricinus *records*

Records of *I. ricinus* ticks in the UK were obtained through the TSS, which has been described in detail previously (Cull et al., 2018; Jameson & Medlock, 2011). Briefly, members of the public, health professionals, veterinary practices, wildlife charities and governmental agencies can submit ticks to the UK Health Security Agency along with a form indicating where and when the tick was acquired and the host the tick was found on. Ticks are identified to species level using morphological keys (Estrada‐Peña et al., 2018; Hillyard, 1996). In this study, we present results for *I. ricinus* only that were found on humans, cats and dogs, which provided sufficient geographical information to map to a 20 km grid. We used records of ticks removed from hosts between 2002 and 2020 and excluded records with recent history of travel overseas in case the tick was imported from another country. As tick numbers submitted through the scheme were low between 2002 and 2012, we grouped these records together and used them as a baseline for possible increases in *I. ricinus* records.

### Analyses

All maps were produced in R (R core team, 2021) using the sf and tmap packages (Pebesma, 2018; Tennekes, 2018). Each of the twelve NUTS1 (Nomenclature of Territorial Units) regions in the UK (Scotland, Northern Ireland, Wales, England: North East, North West, Yorkshire and the Humber, East England, East Midlands, West Midlands, South East, Greater London, South West) were divided into individual grids of 20 km x 20 km (total of 2513 grids), which ranged from 20 grids in Greater London to 960 grids in Scotland. For each year and region, we calculated the percentage of grids that had at least one *I. ricinus* record (number of grids with a record over the total number of grids per region) to compare the change in percentage as well as the cumulative percentage of grids reporting *I. ricinus* over time. To identify possible tick expansion, we calculated, for each region and year, the percentage of grids that had at least one *I. ricinus* record and never reported a record of *I. ricinus* before.

To investigate a temporal change in the distribution of *I. ricinus* ticks, we used two generalized linear mixed effect models (GLMMs) using the lme4 (Bates et al., 2015) package in R.

To identify spatiotemporal changes in the distribution of *I. ricinus*, we used a binomial GLMM with the response variable being the percentage of grids reporting a *I. ricinus* record per year and per NUTS1 region (number of grids with a record vs number of grids without a record). The full model included year (2013–2020), region and the interaction term between year and region were fixed covariates. As to not overfit the model, we grouped NUTS1 regions in fewer categories; North England (North East, North West and Yorkshire & Humber), Central England (West Midlands, East Midlands and East England), South England (South East, South West and Greater London), Wales and Scotland. As Northern Ireland only recorded *I. ricinus* ticks in two years (2017 and 2018), it was removed from the analyses. NUTS1 regions and an observation level random effect, to account for overdispersion (Harrison, 2015) were added as random terms.

To investigate possible tick expansion, we used the percentage of new area covered (grid reporting a tick bite that never reported ticks in previous years) compared to previous years as our response variable in a binomial GLMM. As described above, the full model included year, region and the interaction between year and region as fixed covariates and NUTS1 regions and an observation level random effect were added as random terms.

Model selection was done using the dredge function from the MuMIn package (Barton, 2019) and we selected the model with the smallest AICc.

## RESULTS

Between 2002 and 2020, 4704 records of *I. ricinus* found on humans, cats and dogs with a specific location were submitted through TSS. On average, 487 records (*SD* = 94) are processed per year since 2013 (2002–2012: *n* = 806 records; 2013: *n* = 467; 2014: *n* = 330; 2015: *n* = 446; 2016: *n* = 619; 2017: *n* = 451; 2018: *n* = 616; 2019: *n* = 492; 2020: *n* = 473).

### *Percentage of grids with* I. ricinus *records 2013–2020*

For each region, we calculated the percentage of grids that had at least one record of *I. ricinus*. Overall, 9.2% of the grids in the UK had records of *I. ricinus* ticks submitted to the scheme per year (range: 7.1%–11.2%) (Table 1). In Scotland, 3% of grids reported *I. ricinus* per year (range: 1.9%–5.2%). Regarding Wales, 6.8% of the grids reported ticks (range: 3%–9.6%). All regions in England reported ticks every year with the mean number of grids reporting ticks each year being 14.6% (range: 10.6%–17.6%). The highest number of grid squares with records each year was in South England with 34% (range: 26.2%–41.7%) of grids in South East England recording at least one tick, on average, followed by Greater London with 30% (range: 15%–40%) of grids and South West England with 27.1% (range: 17%–33.5%) of grids. East England reported ticks in 11.8% (range: 8.1%–21.1%) of grids each year, Yorkshire and Humber in 8.1% (range: 4.9%–11.8%) and North West England in 7.9% (range: 4.1%–13.6%). North East England, West Midlands and East Midlands had the lowest coverage with respectively 5.2%, 3.8% and 3.4% of grids reporting *I. ricinus* per year (Table 1).

**TABLE 1:** Percentage of grids with at least one *Ixodes ricinus* record per year for each nomenclature of territorial units for statistics 1 in the UK.

NUTS1	2013	2014	2015	2016	2017	2018	2019	2020	Average
**UK**	**7.8%**	**7.1%**	**9.4%**	**11.1%**	**7.4%**	**11.2%**	**10.0%**	**9.2%**	**9.2%**
**Scotland**	**1.9%**	**3.0%**	**2.3%**	**3.2%**	**2.1%**	**5.2%**	**3.5%**	**2.8%**	**3.0%**
**Wales**	**8.6%**	**5.6%**	**8.6%**	**9.6%**	**6.1%**	**7.6%**	**5.1%**	**3.0%**	**6.8%**
**Northern Ireland**	**0%**	**0%**	**0%**	**0%**	**0.7%**	**2.1%**	**0%**	**0%**	**1.2%**
**England**	**12.3%**	**10.6%**	**15.1%**	**17.6%**	**12.7%**	**17.6%**	**15.6%**	**15.2%**	**14.6%**
North East	1.1%	3.2%	8.6%	6.5%	3.2%	9.7%	6.5%	3.2%	5.2%
North West	4.1%	8.2%	13.6%	8.8%	6.8%	10.2%	4.8%	6.8%	7.9%
Yorkshire and Humber	11.8%	5.6%	10.4%	9.0%	4.9%	6.9%	8.3%	9.0%	8.1%
West Midlands	3.4%	3.4%	3.4%	1.7%	3.4%	4.2%	4.2%	6.7%	3.8%
South East	36.3%	26.2%	32.7%	39.9%	29.2%	41.7%	34.5%	31.5%	34.0%
South West	17.0%	18.4%	25.5%	31.6%	24.5%	33.5%	33.0%	33.0%	27.1%
East Midlands	0.7%	0.7%	3.4%	2.7%	4.0%	4.7%	5.5%	4.0%	3.4%
East England	9.3%	8.1%	9.3%	21.1%	12.4%	12.4%	13.0%	8.7%	11.8%
Greater London	40.0%	25.0%	35.0%	35.0%	15.0%	30.0%	25.0%	35.0%	30.0%

**FIGURE 1:**
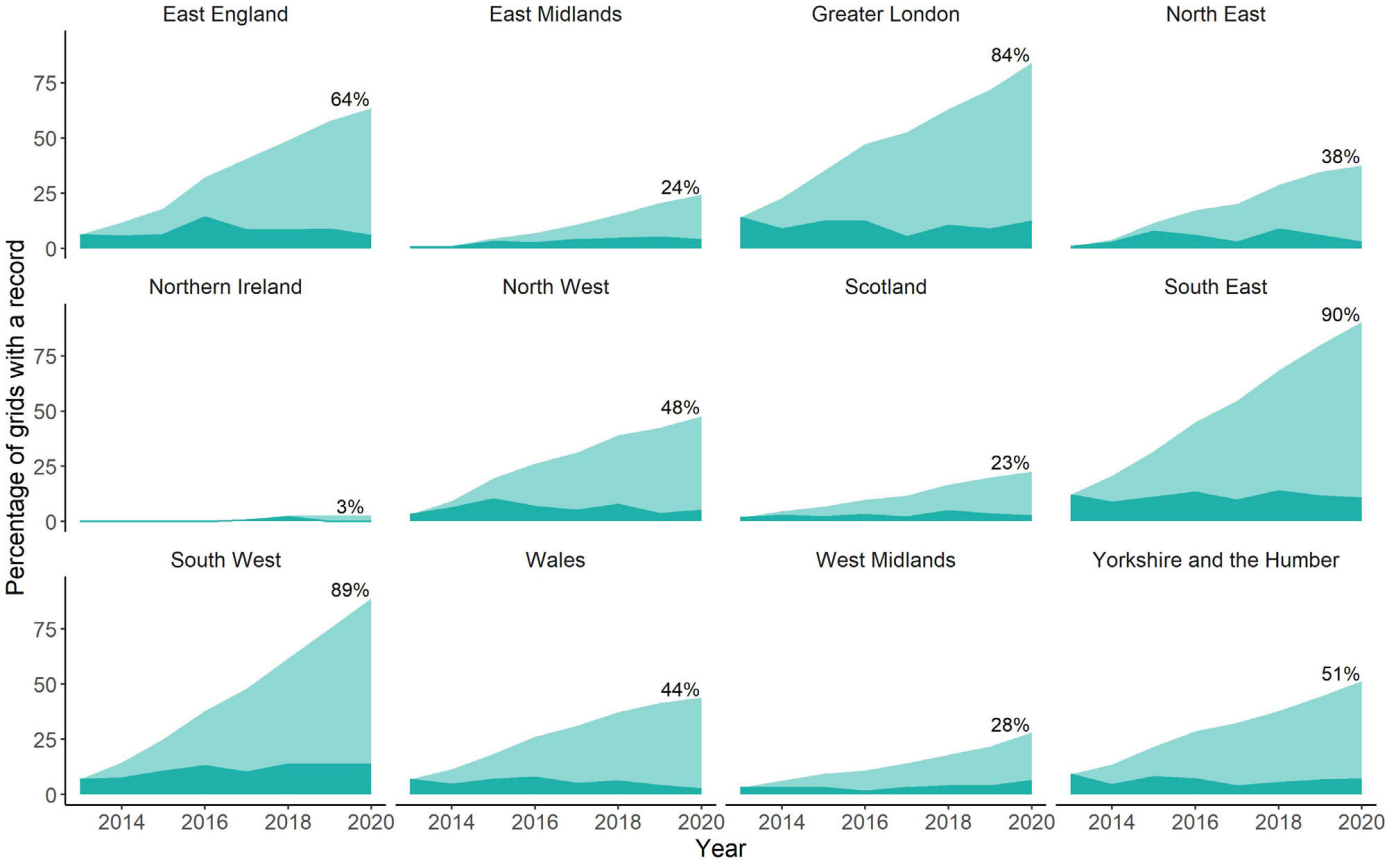
Percentage of grids with at least one record of *Ixodes ricinus* for each nomenclature of territorial units for statistics 1 in the UK between 2013 and 2020. The dark blue colour represents the percentage of grids covered per year and the light blue represents the cumulative percentage of area covered since 2013.

The region with the highest cumulative coverage of *I. ricinus* records is South East England with 90% of grids with a record of *I. ricinus* since 2013, followed closely by South West England with 89% of grids and Greater London with 84% of grids (Figure 1). East England reported ticks in 64% of grids by 2020, followed by Yorkshire and the Humber (51%), North West England (48%), Wales (44%), North East England (38%), West Midlands (28%), East Midlands (24%), Scotland (23%) and Northern Ireland (3%) (Figure 1).

### *Spatiotemporal changes in the distribution of* I. ricinus

**FIGURE 2:**
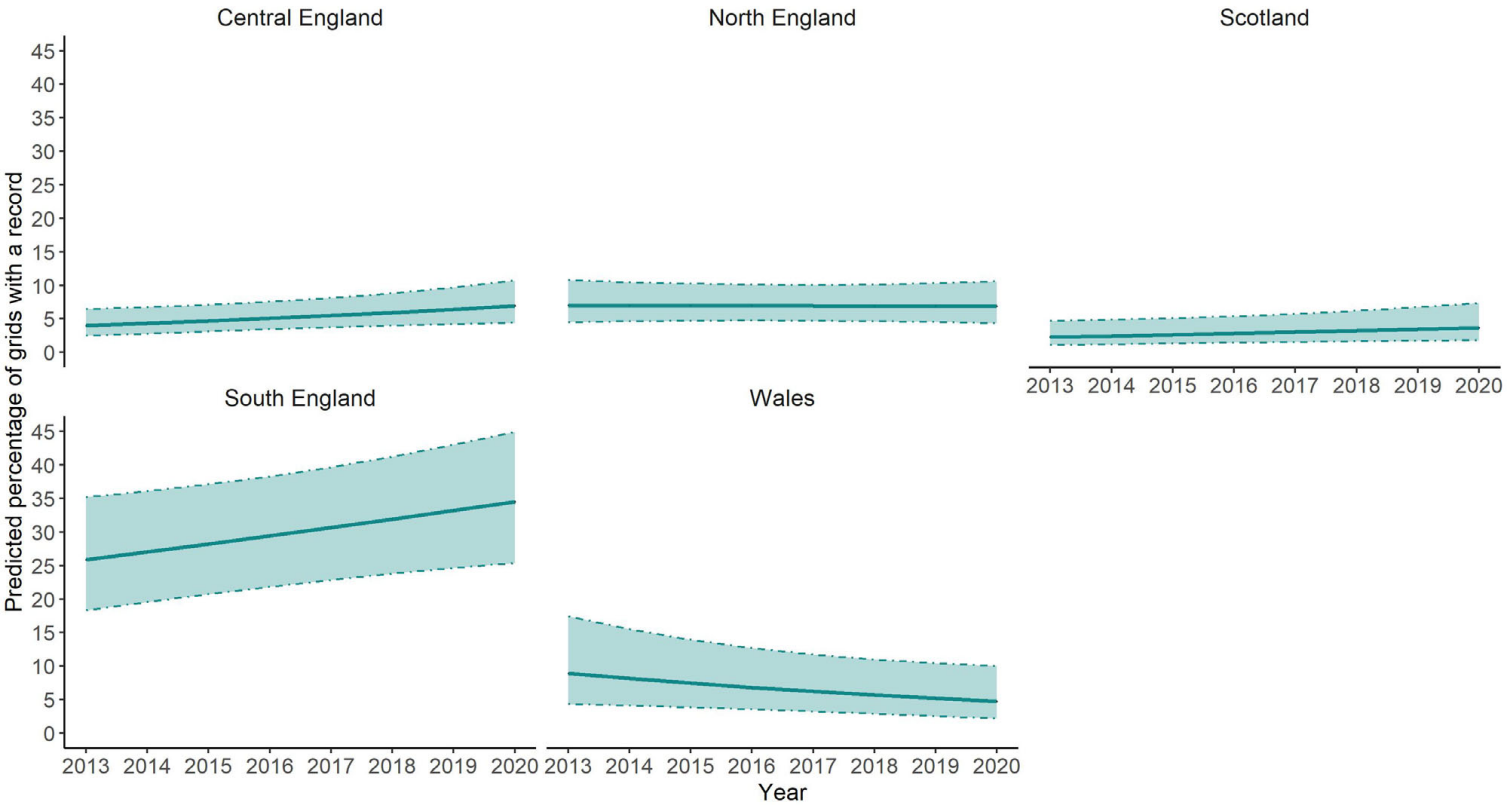
Predicted percentage of grids with an *Ixodes ricinus* record between 2013 and 2020 in Central England, North England, Scotland, South England and Wales. Shaded areas represent 95%CI.

The selected model included region, year and the interaction between region and year (marginal *R* ^2^ = 0.84, conditional *R* ^2^ = 0.99, see Table S1 for results of the model selection). The interaction between region and year was significant in the model (*χ* ^2^[4] = 9.24, *p* = 0.06; see Table S2 for the summary table of the model). Apart from Wales, every region has had an increase in the percentage of grids recording *I. ricinus* every year. South England had, on average, a predicted increase of 1.2% of grids covered each year followed by Central England (0.4%) and Scotland (0.2%). The percentage of grids with an *I. ricinus* record each year generally remained stable in North England with only a 0.02% predicted yearly increase while number of grid squares in Wales had a 0.6% decrease in the percentage of grid recording *I. ricinus* every year (Figure 2, predicted values for each region/year are available in Table S3).

### *Possible* I. ricinus *expansion*

**FIGURE 3:**
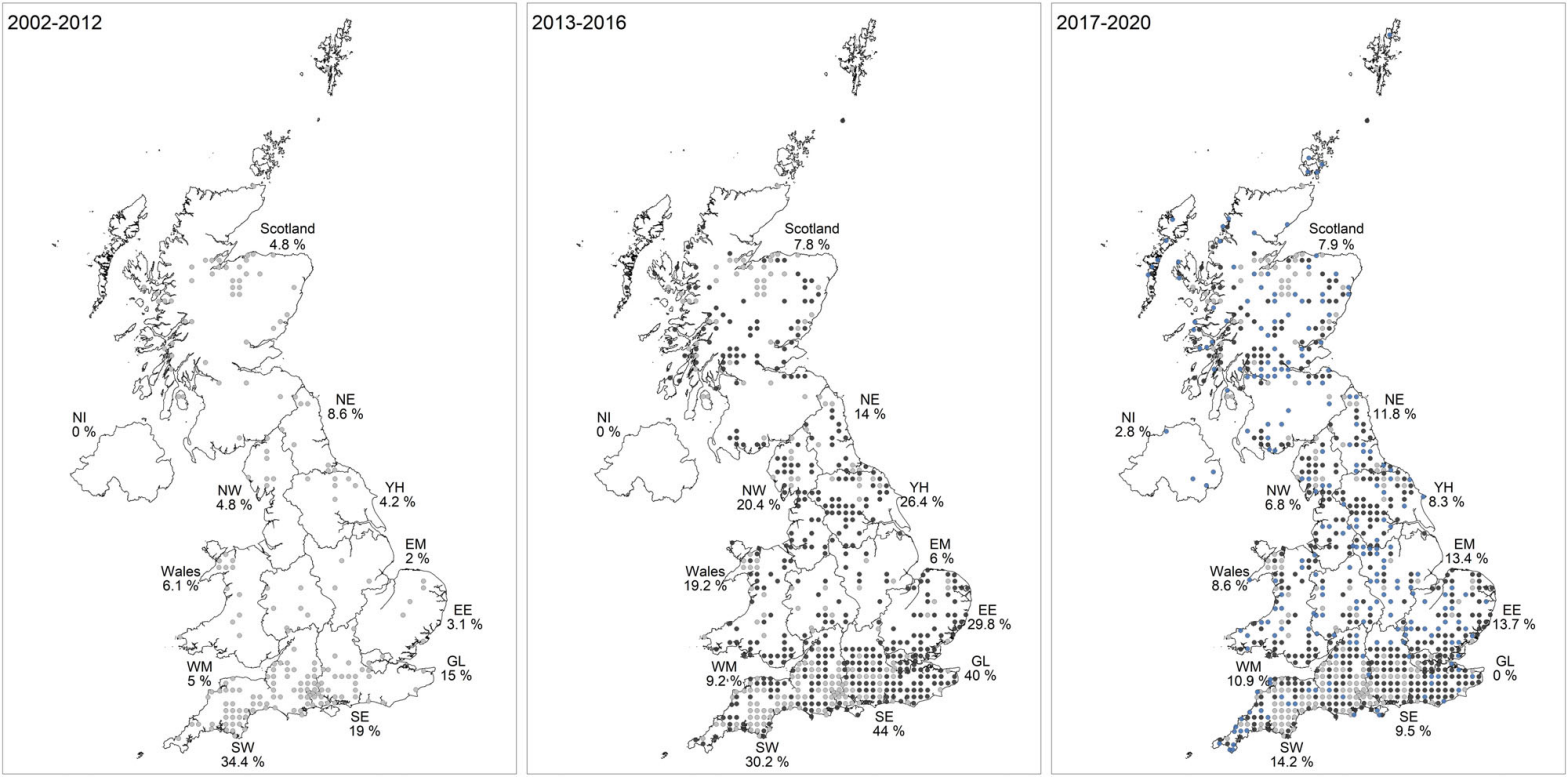
Maps showing the possible expansion of *Ixodes ricinus* records in each region of the UK. The map on the left shows the percentage of grids recording a tick bite between 2002 and 2012 (light grey), the middle map shows the percentage of new grids covered (dark grey) for 2013–2016 and the map on the right shows the percentage of new grids covered (blue) for 2017–2020 compared to previous years. NI: Northern Ireland, NE: North east, NW: North west, YH: Yorkshire and the Humber, WM: West midlands, EM: East midlands, EE: East England, SW: South west, SE: South east, GL: Greater London. Contains ordnance survey data ©crown copyright and database right 2022. Contains National Statistics data ©crown copyright and database right 2022.

We assessed *I. ricinus* expansion in the UK by calculating the percentage of grids that recorded a tick that had never recorded one in previous years (Table 2, Figure 3). Overall, there was a 3.2% increase year on year in coverage in the UK in the number of grids with new records between 2013 and 2020 (range: 1.6%–5.2%). South East England had the highest rate of increase with mean of 6.7% of new grids covered per year with new records, ranging from 0.6% increase in 2020 compared to previous years to 24.4% increase in 2013 compared to 2002–2012. South West England had an expansion of 5.5% per year with three years (2013, 2015 and 2016) having >8% increase on the previous year. East England had a 5.4% yearly increase, with the highest change in 2016 with 11.8%. Greater London had a 5% yearly increase in coverage with the highest change in 2013 with 30% of new area covered. All the other regions reported an increase in *I. ricinus* records, ranging from 1.2% per year in Northern Ireland to 4.3% in Yorkshire and Humber, the latter also reporting an 11.1% increase in coverage in 2013 (Table 2).

**TABLE 2:** Increase in the percentage of grids reporting *Ixodes ricinus* bites compared to previous years (a grid that reported a tick and had never reported one before) for each nomenclature of territorial units for statistics 1 in the UK between 2013 and 2020.

NUTS1	2013^a^	2014	2015	2016	2017	2018	2019	2020	Average increase
**UK**	**5.2%**	**3.1%**	**4.1%**	**3.9%**	**1.8%**	**3.7%**	**2.0%**	**1.6%**	**3.2%**
**Scotland**	**1.3%**	**2.4%**	**1.7%**	**2.5%**	**1.5%**	**3.1%**	**1.7%**	**1.7%**	**2.0%**
**Wales**	**8.1%**	**1.5%**	**5.6%**	**4.0%**	**2.0%**	**3.0%**	**2.0%**	**1.5%**	**3.5%**
**Northern Ireland**	**0%**	**0%**	**0%**	**0%**	**0.7%**	**2.1%**	**0%**	**0%**	**1.2%**
**England**	**8.4%**	**4.2%**	**6.3%**	**5.4%**	**2.2%**	**4.5%**	**2.6%**	**1.8%**	**4.4%**
North East	1.2%	2.2%	7.6%	3.2%	2.2%	6.5%	1.1%	2.2%	3.2%
North West	2.7%	4.8%	8.8%	4.1%	0.7%	2.7%	2.0%	1.4%	3.4%
Yorkshire and the Humber	11.1%	4.2%	8.3%	2.8%	2.8%	2.1%	2.1%	1.4%	4.3%
West Midlands	2.5%	3.4%	2.5%	0.4%	2.5%	1.7%	3.4%	3.4%	2.5%
South East	24.4%	6.0%	7.1%	6.6%	0%	7.1%	1.8%	0.6%	6.7%
South West	8.0%	5.2%	8.5%	8.5%	3.8%	5.7%	3.3%	1.4%	5.5%
East Midlands	0.7%	0.7%	2.7%	2.0%	2.7%	4.0%	4.0%	2.7%	2.4%
East England	8.1%	6.2%	3.7%	11.8%	3.1%	5.6%	2.5%	2.5%	5.4%
Greater London	30.0%	0%	5.0%	5.0%	0%	0%	0%	0%	5.0%

^a^ Increase from the period 2002–2012.

Areas showing recent records of *I. ricinus*, where previously (2002–2016) there had been no reports include for England: parts of the extreme southwest (west and coastal Cornwall), the south midlands (Buckinghamshire, Bedfordshire), the north midlands (Derbyshire, Nottinghamshire, Leicestershire), the Welsh borders (Shropshire, Herefordshire, north Gloucestershire) and the north east (North Yorkshire, Co. Durham); for Wales: parts of the southwest (Cardiganshire, Carmarthenshire); for Northern Ireland (mainly in Co. Down); and for Scotland: central Scotland (including the greater Glasgow area), the Islands (Mull, Outer Hebrides, Orkney), records scattered over the Highlands and Grampian region, and the Lowland areas of Dumfries and Galloway (Figure 3).

### Spatiotemporal changes and possible tick expansion

In terms of detecting potential *I. ricinus* expansion, the selected model included region, year and the interaction between region and year (marginal *R* ^2^ = 0.59, conditional *R* ^2^ = 0.97, see Table S4 for results of the model selection). The interaction between region and year was significant in the model (*χ* ^2^[4] = 17.77, *p* = 0.001). Scotland is the only region that had steady yearly increase in the percentage of new areas covered with, on average, 0.03% of new area recording a *I. ricinus* tick every year (areas which did not previously have a record, see Table S5 for model summary). We obtained this increase rate by calculating the slope of our curve using predicted values for each year. While all the regions had an increase in the numbers of new grids covered every year, there was a decline in the rate in which new grids reported records of *I. ricinus* each year. For example, the rate of increase declined each year in Central England by 0.03%, followed by Wales (decline of 0.57%), North England (decline of 0.59%) and South England (decline of 1.59%) (Table 3).

**TABLE 3:** Predicted percentage [95%CI] of new grids reporting a *Ixodes ricinus* record per year for each region included in the model (grids that never had *Ixodes ricinus* records in previous years).

	2013^a^	2014	2015	2016	2017	2018	2019	2020
Scotland	1.8% [1.0.–3.2]	1.8% [1.1–2.9]	1.8% [1.2–2.7]	1.9% [1.3–2.6]	1.9% [1.4–2.6]	1.9% [1.3–2.8]	2.0% [1.2–3.2]	2.0% [1.1–3.6]
Wales	5.6% [2.9–10.6]	4.7% [2.7–7.9]	3.9% [2.5–6.1]	3.3% [2.2–4.9]	2.7% [1.8–4.2]	2.3% [1.3–3.8]	1.9% [1.0–3.6]	1.6% [0.7–3.5]
Central England	3.4% [2.1–5.3]	3.3% [2.3–4.8]	3.3% [2.4–4.4]	3.2% [2.5–4.1]	3.2% [2.4–4.1]	3.1% [2.3–4.2]	3.1% [2.1–4.4]	3.0% [1.9–4.7]
North England	5.8% [3.8–8.7]	4.9% [3.5–6.8]	4.1% [3.1–5.4]	3.4% [2.7–4.4]	2.9% [2.2–3.8]	2.4% [1.7–3.4]	2.0% [1.3–3.1]	1.7% [1–2.9]
South England	12.6% [8.7–18]	9.5% [7–12.8]	7.1% [5.5–9.1]	5.3% [4.1–6.7]	3.9% [2.9–5.1]	2.9% [2.0–4.0]	2.1% [1.3–3.2]	1.5% [0.9–2.6]

^a^ Increase from the period 2002–2012.

## DISCUSSION

In this study, we aimed to identify spatiotemporal trends in the change in distribution of *I. ricinus* by using data collected through the TSS. According to the TSS data, between 2013 and 2020, 9.2% of grids in the UK reported, on average, a record of *I. ricinus* bite on humans, cats or dogs each year. There was a 3.2% UK wide year on year increase in the percentage of new areas reporting *I. ricinus*, which may suggest an overall increase in the distribution of this species in the UK and could potentially have public and animal health implications.

The areas with the highest percentage of grids covered were in the South and East England (South East, South West, Greater London and East England) while Northern Ireland, East Midlands and Scotland had the lowest percentage of grids reporting a record. These results are in line with those observed by Cull et al. (2018) where older *I. ricinus* records across all host species were taken into account. Scotland, North England, Central England and South England had a yearly increase in the percentage of grids recording *I. ricinus*, which could suggest an expansion in the distribution of *I. ricinus* since 2013. In terms of expansion, all regions (Scotland, Wales, Central England, South England, North England) had an increase in the number of grids with a new record of *I. ricinus* each year, thus suggesting a possible expansion in the range of *I. ricinus*. When we consider this rate of increase however, only Scotland had a steady, positive increase in the rate at which the percentage of new areas covered every year increased. For all other areas, the rate at which the percentage of new areas reporting *I. ricinus* increased each year declined every year since 2013. These results suggest that *I. ricinus* records submitted to the TSS are steadily increasing spatially in all regions of the UK and ticks are being reported in new areas every year (+3.2% per year). Many factors could lead to a possible expansion in tick distribution.

*Ixodes ricinus* are most abundant in woodlands, which provide adequate environmental conditions compared to other less suitable habitats (Estrada‐Peña, 2001; Pfäffle et al., 2013) so spatial expansion could be linked to increase in woodland cover and connectivity, which has been widely promoted in the UK (Forestry‐Commission, 2022). Indeed, the surface covered in forest/woodland in the UK has increased by 6.2% between 1998 (12% of the UK covered) and 2020 (13% of the UK covered). In England the area covered by woodlands has increased by 0.3% per year since 1999 while it has increased by 0.2% in Wales, 0.6% in Scotland and 1.7% in Northern Ireland every year (Ward, 2021). In terms of area, forests increased by 134 km^2^ per year since 1998 in the UK, representing 0.06% of the country's surface (England: 34 km^2^ or 0.03%, Wales: 5 km^2^ or 0.02%, Scotland: 78 km^2^ or 0.1%, Northern Ireland: 16 km^2^ or 0.12%) (Ward, 2021). This increase in woodland/forest across the UK might have created additional suitable habitat for *I. ricinus* and it would be interesting to compare new areas where tick bites have been reported with areas where forests have also expanded.

Whilst reforestation and afforestation can increase the area providing suitable environmental condition for *I. ricinus*, it also provides habitats for deer, which are known to sustain high tick densities (Gandy et al., 2021; Gilbert et al., 2012). Deer densities have steadily increased since the 1980s for all species (Red deer *Cervus elaphus*, Artiodactyla: Cervidae, L.; roe deer *Capreolus capreolus*, Artiodactyla: Cervidae, L.; fallow deer *Dama dama*, Artiodactyla: Cervidae, L.; muntjac deer *Muntiacus reevesi*, Artiodactyla: Cervidae, Ogilby) and, while red deer and fallow deer populations have stabilized since the mid‐2000s, roe deer densities have increased until the mid‐2010s, when they started to plateau (GWCT, 2016). Muntjac deer population has increased 15‐fold since the 1980s and is still rising (GWCT, 2016). Since deer are tick reproduction hosts and drive tick density, the possible expansion of *I. ricinus* observed could be linked to an increase in deer densities across the UK (Medlock et al., 2013; Sonenshine, 2018).

Several studies have investigated the effect of climate change on tick densities. For instance, the spread of *I. ricinus* northwards in Sweden has been linked to milder winters (Jaenson et al., 2012; Lindgren et al., 2000; Lindgren et al., 2006). In the case of our study, climate change is unlikely to have caused expansion over such a short period of time. However, increase in temperature over the next few decades could affect tick questing activity, with *I. ricinus* likely to start questing earlier in the spring (Medlock et al., 2013).

While investigating environmental drivers that could affect tick distribution is crucial, other factors could lead to an increase in the number of tick bites reported. For instance, an increase in human population could result in higher probabilities of people and companion animals encountering a tick. Since 2012, the human population has increased in all regions of the UK, ranging from 0.36% to 1.07%. In the South England, the regions recording the highest increases in 20 km grids with *I. ricinus* records, had at least 0.73% increase in human population (South West: 0.75%, South East: 0.73%, East England: 0.77%, Greater London: 1.07%) (ONS, 2020). An increase in awareness about ticks, and the TSS might have also biased our results, as more people might be submitting records through the scheme if levels of awareness are increasing. The number of individual recorders has increased from 205 in 2013 to 354 in 2016 but has remained steady since (between 268–354 recorders per year in 2016–2020) so the recent rise in tick records submitted is unlikely to be due to an increase in recorders or awareness alone. This possible expansion may have been partly responsible for the increasing annual incidence of Lyme borreliosis reported in the UK, particularly in the South (Cairns et al., 2019). Other factors for increasing incidence could also include increased awareness of the disease among health care providers, as well as human behavioural changes.

In this study, we report a possible expansion in the distribution of *I. ricinus* bites on humans, dogs and cats in the UK. While every region had a year on year increase in the percentage of new areas reporting *I. ricinus* records, there was a steady decline in the rate at which the percentage of new areas reporting *I. ricinus* increased each year, except for Scotland. Many reasons could explain this waning, especially in the South England where a high percentage of grids are already reporting records.

While the data show an increase in the total area covered as well as new areas reporting tick bites every year, we could not conduct statistical analyses at the NUTS1 level and we had to group NUTS1 by wider regions due to the low sample size. It would be interesting to compare the change in the distribution of *I. ricinus* with land use changes and deer densities to better understand the drivers of changes in *I. ricinus* distribution. We used data from a passive surveillance scheme, which has its limitations; ticks are sent by individuals or groups that are aware of the TSS and interested in participating. However, other individuals in the country might encounter ticks and not send them through the TSS. Regardless, it is important to continue passive tick surveillance through TSS as the data gathered can help understand vector distribution and potential shifts and facilitate assessments of the impact of climate and environmental change and their utility as indicators. While this study focuses on *I. ricinus*, as it is the vector of many human and animal pathogens, other tick species in the UK are vectors of diseases‐causing pathogens. Using data collected through TSS, Medlock et al. (2017) and Medlock et al. (2018) showed how two other tick species (*H. punctata* and *D. reticulatus*) may be changing distribution in some parts of South England. Longer‐term monitoring would provide a more robust dataset to start making assessments on the possible impacts of climate change, which are not currently possible.

To conclude, records of *I. ricinus* in the UK reported on humans, cats and dogs have steadily increased since 2012 and the area reporting new records is expanding every year. Incorporating additional datasets for analysis alongside the TSS data, such as land use change, deer density, human behaviour and Lyme borreliosis incidence, may help to further our understanding of possible expansion of important tick species in the UK, as well as the impact on human health.

## AUTHOR CONTRIBUTIONS

Sara L. Gandy conducted statistical analyses and lead the writing of the manuscript. Kayleigh M. Hansford manages the delivery of the Tick Surveillance Scheme and coordinated the writing of this publication with Sara L. Gandy and Jolyon M. Medlock. Jolyon M. Medlock conceived the idea and developed this with Sara L. Gandy and Kayleigh M. Hansford. All authors contributed to and approved the final version of the manuscript.

## CONFLICT OF INTEREST

The authors report no potential conflict of interest.

## Supporting information


**Appendix S1** Supporting Information

## Data Availability

The data that support the findings of this study are available from the corresponding author upon reasonable request.
