# Supplementary material for: Possible expansion of Ixodes ricinus in the United Kingdom identified through the Tick Surveillance Scheme between 2013 and 2020
Source: Med Vet Entomol. 2022 Oct 14;37(1):96–104. doi: 10.1111/mve.12612 (PMC10092138; doi:10.1111/mve.12612)
Supplement: Supplementary file 1 — Appendix S1 Supporting Information [file MVE-37-96-s001.docx]

**Supplementary Information**

Gandy et al., 2022. Possible expansion of *Ixodes ricinus* in the United Kingdom identified through the Tick Surveillance Scheme between 2013 and 2020

Table S1: Table summarising the output from the dredge function (MuMIn package) showing the different models tested for the model focussing on the effects of region and year on the percentage of grids recording a tick bite. The first model, with the lowest AICc was selected.

| Intercept | Region | Year | Region * Year | df | LogLik | AICc | delta AICc |
| --- | --- | --- | --- | --- | --- | --- | --- |
| -0.334 | + | 0.08 | + | 12 | -264.6 | 547.1 | 0 |
| -0.084 | + | 0.04 |  | 8 | -260 | 548.2 | 1.1 |
| -2.875 | + |  |  | 7 | -266.9 | 549.2 | 2.05 |
| -2.507 |  | 0.04 |  | 4 | -275.9 | 560.3 | 13.14 |
| -2.298 |  |  |  | 3 | -278.2 | 562.6 | 15.49 |

Table S2: Outputs from the generalized linear mixed effect model explaining the effects region and year on the percentage of grids recording a tick bite.

|  | Estimate | Std. Error | Z-value | P-value |
| --- | --- | --- | --- | --- |
| Intercept | -3.33 | 0.3 | -10.97 | <0.001 |
| **Region** |  |  |  |  |
| North England vs Central England | 0.75 | 0.42 | 1.81 | 0.07 |
| Scotland vs Central England | -0.56 | 0.52 | -1.07 | 0.28 |
| South England vs Central England | 2.16 | 0.4 | 5.47 | <0.001 |
| Wales vs Central England | 1.2 | 0.55 | 2.19 | 0.03 |
| **Year** | 0.08 | 0.04 | 2.18 | 0.03 |
| **Year * Region** |  |  |  |  |
| Year * North England vs Central England | -0.09 | 0.05 | -1.64 | 0.1 |
| Year * Scotland vs Central England | -0.01 | 0.06 | -0.2 | 0.84 |
| Year * South England vs Central England | -0.02 | 0.05 | -0.5 | 0.62 |
| Year * Wales vs Central England | -0.18 | 0.07 | -2.65 | 0.008 |

Table S3: Precited percentage of grids reporting an *Ixodes ricinus* record per year for each region included in the model.

| Year | Scotland | Wales | Central England | North England | South England |
| --- | --- | --- | --- | --- | --- |
| 2013 | 2.29%  [1.1-4.69] | 8.90%  [4.32-17.44] | 4.00%  [2.48-6.47] | 7.00%  [4.50-10.80] | 25.90%  [18.40-35.20] |
| 2014 | 2.45%  [1.22-4.87] | 8.15%  [4.11-15.53] | 4.36%  [2.79-6.75] | 7.00%  [4.60-10.50] | 27.00%  [19.60-36.10] |
| 2015 | 2.63%  [1.34-5.08] | 7.45%  [3.85-13.96] | 4.72%  [3.11-7.10] | 7.00%  [4.70-10.20] | 28.20%  [20.70-37.10] |
| 2016 | 2.81%  [1.45-5.37] | 6.82%  [3.55-12.7] | 5.10%  [3.42-7.54] | 6.90%  [4.73-10.08] | 29.40%  [21.90-38.30] |
| 2017 | 3.01%  [1.56-5.73] | 6.23%  [3.22-11.72] | 5.510%  [3.72-8.10] | 6.90%  [4.70-10.05] | 30.60%  [22.90-39.70] |
| 2018 | 3.22%  [1.66-6.17] | 5.69%  [2.87-10.97] | 5.96%  [3.99-8.81] | 6.90%  [4.60-10.10] | 31.90%  [23.80-41.30] |
| 2019 | 3.45%  [1.74-6.72] | 5.19%  [2.52-10.4] | 6.43%  [4.23-9.67] | 6.90%  [4.50-10.30] | 33.20%  [24.60-43.00] |
| 2020 | 3.69%  [1.81-7.37] | 4.74%  [2.18-9.98] | 6.94%  [4.4-10.72] | 6.90%  [4.40-10.60] | 34.50%  [25.40-45.00] |

Table S4: Table summarising the output from the dredge function (MuMIn package) showing the different models tested for the model focussing on the effects of region and year on the percentage of new grids recording a tick bite (grids that never recorded a tick bite before). The first model, with the lowest AICc was selected.

| Intercept | Region | Year | Region * Year | df | LogLik | AICc | delta AICc |
| --- | --- | --- | --- | --- | --- | --- | --- |
| -3.323 | + | -0.02 | + | 12 | -219.1 | 466.4 | 0 |
| -2.661 | + | -0.14 |  | 8 | -228 | 473.9 | 7.43 |
| -2.606 |  | -0.15 |  | 4 | -233.3 | 475 | 8.59 |
| -3.478 | + |  |  | 7 | -237.2 | 499.8 | 23.36 |
| -3.418 |  |  |  | 3 | -241.9 | 490.1 | 23.73 |

Table S5: Outputs from the generalized linear mixed effect model explaining the effects region and year on the percentage of new grids recording a tick bite (grids that never recorded a tick bite before).

|  | Estimate | Std. Error | Z-value | P-value |
| --- | --- | --- | --- | --- |
| Intercept | -3.32 | 0.34 | -9.75 | <0.001 |
| **Region** |  |  |  |  |
| North England vs Central England | 0.9 | 0.47 | 1.9 | 0.06 |
| Scotland vs Central England | -0.73 | 0.56 | -1.3 | 0.19 |
| South England vs Central England | 2.02 | 0.46 | 4.37 | <0.001 |
| Wales vs Central England | 0.78 | 0.61 | 1.43 | 0.15 |
| **Year** | -0.02 | 0.06 | -0.3 | 0.77 |
| **Year * Region** |  |  |  |  |
| Year * North England vs Central England | -0.16 | 0.08 | -1.99 | 0.05 |
| Year * Scotland vs Central England | 0.03 | 0.09 | 0.36 | 0.72 |
| Year * South England vs Central England | -0.3 | 0.08 | -3.66 | <0.001 |
| Year * Wales vs Central England | -0.17 | 0.11 | -1.57 | 0.12 |
